# Renin–Angiotensin System in Liver Metabolism: Gender Differences and Role of Incretins

**DOI:** 10.3390/metabo12050411

**Published:** 2022-05-03

**Authors:** Zainab Mastoor, Yolanda Diz-Chaves, Lucas C. González-Matías, Federico Mallo

**Affiliations:** Biomedical Research Centre (CINBIO), University of Vigo, 36310 Vigo, Spain; zainab.mastoor@uvigo.es (Z.M.); yolandadiz@uvigo.es (Y.D.-C.); lucascgm@uvigo.es (L.C.G.-M.)

**Keywords:** renin-angiotensin system, RAS, liver metabolism, GLP-1, perinatal undernutrition

## Abstract

The impaired hepatic lipids and carbohydrates metabolism result in various metabolic disorders, including obesity, diabetes, insulin resistance, hyperlipidemia and metabolic syndrome. The renin–angiotensin system (RAS) has been identified in the liver and it is now recognized as an important modulator of body metabolic processes. This review is intended to provide an update of the impact of the renin–angiotensin system on lipid and carbohydrate metabolism, regarding gender difference and prenatal undernutrition, specifically focused on the role of the liver. The discovery of angiotensin-converting enzyme 2 (ACE2) has renewed interest in the potential therapeutic role of RAS modulation. RAS is over activated in non-alcoholic fatty liver disease (NAFLD) and hepatocellular carcinoma. Glucagon-like peptide-1 (GLP-1) has been shown to modulate RAS. The GLP-I analogue liraglutide antagonizes hepatocellular steatosis and exhibits liver protection. Liraglutide has a negative effect on the ACE/AngII/AT1R axis and a positive impact on the ACE2/Ang(1-7)/Mas axis. Activation of the ACE2/Ang(1-7)/Mas counter-regulatory axis is able to prevent liver injuries. Angiotensin(1-7) and ACE2 shows more favorable effects on lipid homeostasis in males but there is a need to do more investigation in female models. Prenatal undernutrition exerts long-term effects in the liver of offspring and is associated with a number of metabolic and endocrine alterations. These findings provide a novel therapeutic regimen to prevent and treat many chronic diseases by accelerating the effect of the ACE2/Ang1-7/Mas axis and inhibiting the ACE/AngII/AT1R axis.

## 1. Introduction

Liver diseases are major causes of morbidity and mortality globally [1]. The growing epidemic of overweight and obesity is one of the leading causes of an increased prevalence of metabolic diseases, including impaired lipids and glucose homeostasis [2]. In fact, obesity and type 2 diabetes mellitus are commonly associated with fat deposition in the liver and two of the risk factors for hepatic fibrosis [3]. Experimental and clinical studies have shown that both axes of the RAS, as detailed below, are very important in the regulation of general and liver metabolism and may take part in the pathogenesis of liver metabolism and diseases [1]. In fact, RAS imbalance appears to promote hepatic fat depot, inflammation and fibrogenesis [3]. The activation of the fibrosis process with extracellular matrix deposition in the liver may occur by recurrence of the inflammatory injury resulting to scar formation, and scarring may progress from fibrosis to liver cirrhosis [4]. The pathological characteristics of chronic liver diseases include oxidative stress and increase in inflammatory and pro-fibrosis markers, and RAS is linked with all these processes [1,5].

**Figure 1:**
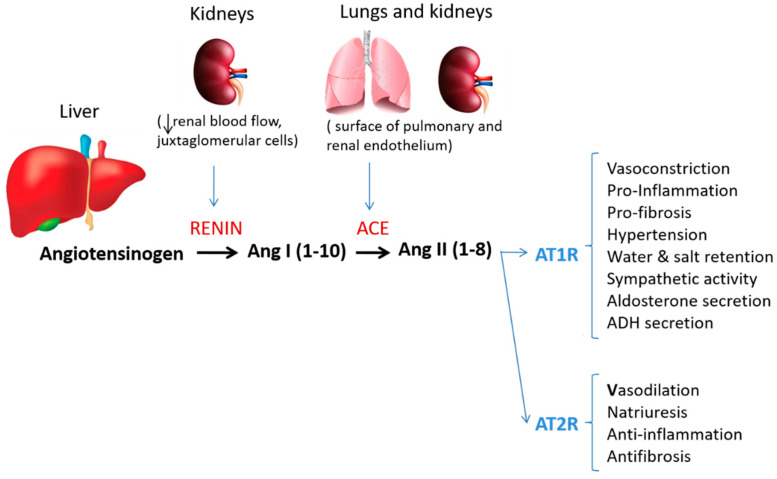
Classical RAS components. From angiotensinogen a cascade of enzyme generates different active peptides, which more relevant is AngII. AngII might activate 2 different receptors, AT1R and AT2R, having counter-regulatory effects in vessel tone, inflammation, and body fluid control.

**Figure 2:**
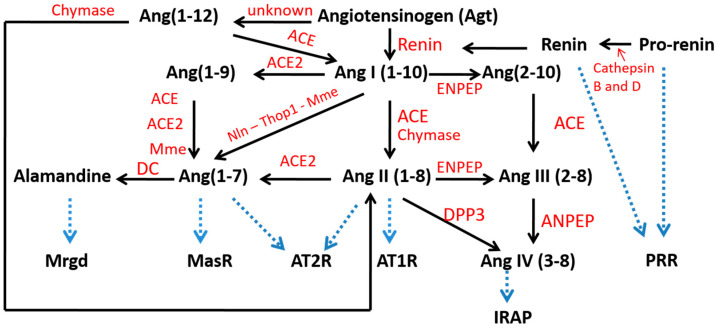
Non-classical RAS components. Renin is activated by its binding to the PRR receptor and enzyme cathepsin B and D. Renin cleaves the precursor protein angiotensinogen and enzymatic cascade of formed angiotensin peptides that bind with the different receptors T1R, AT2R, IRAP, MrgD and MasR and exert their effects. Angiotensin-converting enzyme (ACE) and angiotensin-converting enzyme 2 (ACE2), neurolysin (Nln), neprilysin (Mme), thimetoligopeptidase (Thop 1), angiotensin I receptor (AT1R), insulin-regulated aminopeptidase (IRAP), Mas-related G-protein coupled receptor member D (Mrgprd), pro-renin receptor (PRR), aminopeptidase A (ENPEP), aminopeptidase N (ANPEP), decarboxylation (DC)).

The renin–angiotensin system (RAS) is considered a hormonal system mainly responsible for blood pressure control and hydroelectrolyte balance [1]. In the last three decades, the relevance of the RAS has been reinforced by the uncovering of new more specific receptors, new enzymes and alternative pathways of processing angiotensin II (AngII), differentially expressed in specific tissues and organs. The findings regarding Ang(1-7) that opposes the vasoconstrictive, proliferative, pro-fibrotic, and pro-inflammatory effects mediated by AngII have supported the fact that the RAS is composed of two branches [6]. The first consists of the angiotensin-converting enzyme (ACE), that converts AngI to AngII, and AngII mediates its biological effect by the angiotensin type 1 (AT1) receptor. Up to here, these elements constitute the bases of the classical RAS (as shown in Figure 1). The second pathway is initiated by the hydrolysis of AngII by ACE2 enzyme to Ang(1-7), which owns its specific receptor, the Mas receptor. Ang(1-7) exerts the vasodilatory, anti-proliferative, anti-fibrotic, and anti-inflammatory effects as opposed to AngII [6]. In addition, the discovery in recent years of new enzymes supporting alternative synthesis pathways and new end-products, represented by other small peptides as amantadine and AngIV with their respective specific receptors and biological activities, amplified the wingspan of RAS to a non-classical more complex model (Figure 2).

Finally, there are very deep gender differences in liver metabolism and hormonal responses. Those differences may become conditioned by events occurring even before birth. Gestational undernutrition decreased peripheral insulin sensitivity and increased hepatic lipid accumulation in lambs, mice, rats, and humans [7]. Disease symptoms, their severity, and treatments will also be different between genders; thus, there is a need to learn about disease mechanisms in both genders [8].

## 2. Metabolic Role of Liver in Lipid and Carbohydrate Metabolism

The liver is the largest parenchymal viscera of vertebrates, lying just below the diaphragm. The liver takes up nutrients directly from the portal blood containing also insulin. The liver is a highly specialized tissue consisting of mostly hepatocytes that process and detoxify various metabolites, synthesize proteins and regulate a wide variety of biochemical reactions necessary for digestion and general metabolism. The liver is thought to be responsible for up to 500 separate functions, usually in combination with other systems and organs, and it has a remarkable great regeneration capability against injury [9].

The liver is the key regulatory organ for the modulating of lipid metabolism, maintaining the homeostasis and energy balance [10]. It is a metabolically active organ, where carbohydrates may be transformed into cholesterol (CL) and fatty acids (FA), which can be esterified into TG for storage, or for secretion into lipoproteins that will circulate in blood to be used by other tissues [11]. The liver is a hub of fatty acid synthesis and lipid circulation.

**Figure 3:**
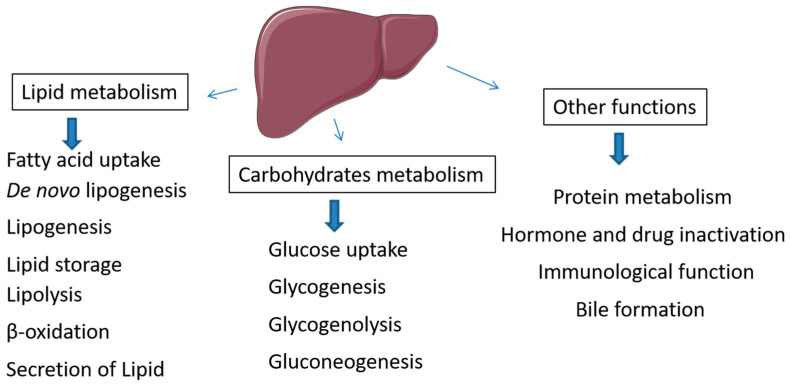
Metabolic role of liver in lipid and carbohydrate metabolism. Liver plays key role in regulation of metabolic processes of lipogenesis, lipolysis, lipid secretion, glycogenesis, glycogenolysis and gluconeogenesis.

It is very well known the central role of the liver in maintaining blood glucose levels. Following the insulin-dependent uptake of glucose, excess glucose can be stored within the liver or muscles as glycogen and glycogen can be degraded to release glucose in times of fasting or during exercise. The liver maintains glucose level by supporting key metabolic processes as glycogenesis, glycogenolysis, and gluconeogenesis (Figure 3). The liver is also the major site of conversion of carbohydrates into lipids, but also of producing ketone bodies from lipids as alternative energy substrate to carbohydrates. In the postprandial period, the islet’s insulin targets first the liver to promote glucose and fatty acid uptake to address lipogenesis, and suppresses lipolysis. Uptake of circulating lipids, lipogenesis, and lipolysis and hence adipocyte metabolism depend on insulin circulating levels and the balance between anabolism and catabolism. These processes are regulated by endocrine messengers and the sympathetic nervous system. When nutrient intake exceeds energetic expenditure during a prolonged period, the liver and muscles store the energy excess in the form of lipids and engender adipose tissue growth and persistent hyperglycemia. Obesity is often associated with elevated insulin secretion from pancreatic β cells [12] and hyperinsulinemia effects in organs will promote increased deposition of fat in liver and adipose tissue and drive current obesity complications.

## 3. Local Hepatic RAS

The renin–angiotensin system is a complex network of circulating molecules, enzymes and receptors, expressed in multiple organs. The liver produces and secretes angiotensinogen as depicted in Figure 1, which is the precursor of the angiotensins, the active peptides constituting the RAS. Classically, the final effectors of the system are small peptides, from which angiotensin-II (AngII) was considered the key factor accounting for most of the biological activities attributed to this group of molecules [13]. The final production of AngII involves different substrates and several enzymes produced in the kidney, liver and lungs (see above Figure 2). The primal precursor of this chain is angiotensinogen, produced and released to the circulation from the liver. The multiple key other components of the RAS have been also identified in the liver, as in many different organs, that amplify our view that some biological effects may depend on the local tissue expression of the different components of RAS. There is evidence of complete local RAS in mice liver of both normal and cancerous cells [3,6,14,15]. The liver is the primary source of angiotensinogen for central RAS in the circulation [3,14,16]. According to one study, angiotensin II is mainly produced by hepatic Kupffer’s cells and hepatocytes [9]. In addition, the *renin* gene has been identified in hepatocytes [17] but its expression appears to be specifically repressed in vivo [18]; thus, its biological role as potential activator of the pro-renin receptor remains to be elucidated [18]. Local RAS in liver has been particularly related to regeneration in liver fibrosis, injury and apoptosis [19]. The two RAS axis recognized both in circulation and locally in the liver are more activated in chronic liver diseases and liver injury [6]. RAS is frequently activated in chronic liver diseases, where AII is a key factor for promoting fibrosis [16].

In the heart, elevation of classical RAS may induce cardiac hypertrophy, fibrosis, heart failure (HF) and atrial fibrillation (AF) while the non-classical RAS exerts cardioprotective effects [20]. Some researchers suggest that hepatic fibrosis is associated with increased AngII/AT1, and on the contrary antagonized by Ang(1-7), which plays a protective role [3]. It has been demonstrated that Ang(1-7) is upregulated in human liver disease having marked anti-fibrotic activity. In a cirrhotic human liver and in rat liver injury, the activation of the ACE2/Mas receptor branch promotes hepatic conversion of angiotensin II to Ang(1-7), leading to beneficial effects in liver diseases and repairing after injuries [3]. One study has shown that the blockade of RAS is related to tumor growth inhibition in colorectal cancer (CRC) and liver metastasis in animal models [15].

### 3.1. Angiotensinogen Regulation in Liver

Angiotensinogen (AGT) is a precursor protein of all angiotensin peptides, produced and secreted mainly by the liver and cleaved by renin to angiotensin I. Its regulation and function are complex. The interest in AGT has grown as it is rate-limiting in the generation of angiotensin I [21]. AGT synthesis is stimulated by glucocorticoids, thyroid hormones and estrogens. These hormones increase the transcription of the AGT gene and AGT mRNA concentration [22].

Various components of renin–angiotensin system (RAS) involved in feedback control of AGT production, renin and AngII are associated with feedback regulation of AGT; thus, renin may inhibit AGT release rate and AngII stimulates it [21]. The elevated plasma renin concentration may have direct inhibitory action on AGT synthesis in hepatocytes or may inactivate an endogenous circulating angiotensinogen-stimulating factor [19]. AngII is also one of the factors that regulate AGT level. The infusion of AngII to experimental animals elevates the AGT levels in liver, by inhibiting the intracellular adenylyl cyclase pathway-cAMP [22,23].

In addition, some other humoral factors such as cytokines may have a role in regulating AGT expression in the liver at specific conditions, since interleukin-6 (IL-6) increased the AGT expression during liver regeneration depending on the JAK/STAT3 and JAK/p38/NF-kB signaling pathways [24].

### 3.2. Pro-Renin Receptor in Liver Metabolism

Pro-renin is the biochemical precursor of renin. The pro-renin receptor (PRR) is a transmembrane protein of 350 amino acids encoded by the ATP6AP2 gene in human beings [25] that regulates tissue RAS [26]. Renin and pro-renin both bind the PRR to induce non-proteolytic activation which increases the catalytic efficiency for AngII production in tissues and initiates AngII-independent intracellular signaling [27]. Renin is the only rate-limiting enzyme of the RAS, and it induces the conversion of AGT to AngI [26]. The binding of pro-renin to cell surface PRR also ultimately leads to local generation of AngI and AngII [26].

Pro-renin regulates cellular lipid levels in the liver, including triglycerides and cholesterol, both markedly increased in the presence of renin–prorenin.

**Figure 4:**
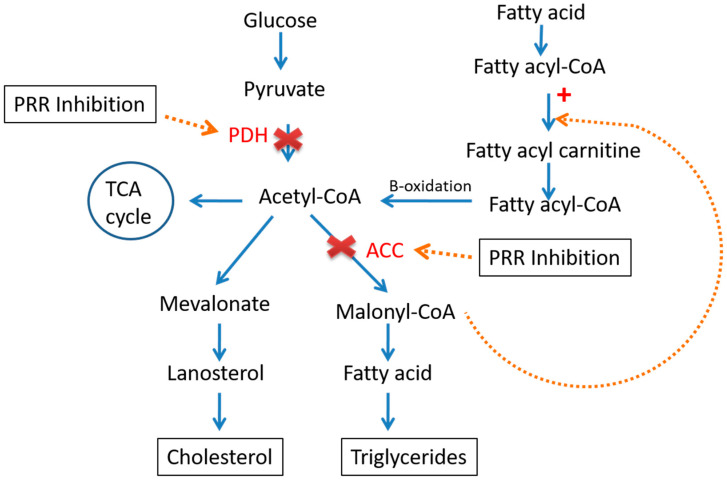
Role of pro-renin receptor (PRR) in glucose and lipid metabolism. Inhibition of PRR (N-acetyl-galactosamine PRR antisense oligonucleotide (G-PRR) used to inhibit PRR expression) decreases pyruvate dehydrogenase (PDH) and acetyl-CoA carboxylase (ACC), reduction in ACC in turn decreases the malonyl Co A that limits the FA synthesis.

In fact, it has been found that the PRR inhibition tends to decrease the level of acetyl-CoA carboxylase (ACC) and pyruvate dehydrogenase (PDH), the crucial enzymes involve in fatty acid (FA) synthesis, which leads to the limiting of FA biosynthesis [28]. In addition, decreased levels of malonyl-CoA will increase the supply of fatty acyl-CoA to the mitochondria; on the other hand, increased fatty acid oxidation will indeed increase the production of acetyl-CoA, which will go into the TCA cycle or ketogenesis pathway but not to fatty acid synthesis [28] (Figure 4).

Interestingly, renin inhibition improves insulin sensitivity, glucose tolerance, and insulin secretion in male rodent models of hypertension, diabetes, obesity, and metabolic syndrome [27]. PRR expression level is elevated in human and mouse fibrotic livers besides heart and lung fibrosis, and downregulation of PRR prevents the liver fibrosis [25]. The knockdown of PRR expression in the liver improves fibrosis [25].

### 3.3. Angiotensin-Converting Enzyme (ACE) and Angiotensin II

Angiotensin-converting enzyme (ACE) is a main component of RAS. This dicarboxypeptidase removes two C-terminal amino acids from AngI and generates active peptide AngII as an end product of ACE [23,26]. AngII has two receptors, the AngII type I receptor (AT1R) and the AngII type II receptor (AT2R). The number of AT1R after birth elevates the number of AT2R, making the effects of AngII mainly mediated through AT1R [26]. Both receptors are G-protein-coupled receptors with seven transmembrane domains [29]. The major biological actions of AngII are mediated by AT1R [1]. AngII acts at cell surface type I G protein-coupled receptors (AT1R) to induce vasoconstriction, pro-inflammatory conditions, oxidative stress [1,27,29] and insulin resistance in the liver [27].

Ang II also binds cell surface type II receptors (AT2R) to restrain AT1R-mediated actions, although these receptors are more limited in tissue expression and affinity [27]. The increased expression of ACE in areas of active fibrogenesis as compared to control animals indicate that classical components of RAS and ACE/AngII/AT1R must have a role in the pathogenesis of liver fibrogenesis [3,26]. The liver ACE/ANG II axis is mainly activated in patients with chronic liver disease and plays important roles in hepatic fibrosis and portal hypertension [13,26]

Liver fibrosis progression depends on interactions among injured hepatocytes, activated inflammatory cells and hepatic myofibroblasts [1]. Hepatic stellate cells (HSCs), also called lipid storage cells, are mainly responsible for excessive deposition of connective tissue components including type I collagen in response to liver injury [5], AT1R involved in HSC activation and collagen deposition in chronic liver disease [1]. Portal fibroblasts, circulating fibroblasts, and bone-marrow-derived cells are involved in hepatic fibrogenesis, but the most pivotal cell type is HSCs, which secrete collagen types I and III [30].

AngII induces HSC proliferation and upregulates transforming growth factor (TGF-β1) in vitro [1]; TGF-β1 plays a dominant role in the development of fibrosis [6]. In liver injuries, ACE and AT1R levels are increased, and are highly expressed by activated HSCs both in vivo and in vitro [30].

ACE/AngII/AT1 axis inhibitors block AngII production and its effects, preventing the development of hepatic fibrosis and other liver diseases [3,26,31]. Liver injuries in AT1 knockout mice present reduced inflammation and fibrosis [3]. ACE inhibitors and ARBs are also used cardioprotectively for hypertension treatment in obese and type II diabetic patients [25]. ACE inhibitors and ARBs show cardioprotective effects by the inhibition of atrial fibrosis and inflammation, the prevention of electrical cardiac remodeling, and the epicardial adipose tissue’s modulation [20].

ACE inhibitor and captopril effectively reduced TGF-β1 and collagen gene expression, reduced collagen deposition, and delayed the progression of hepatic fibrosis [32]

ACE activity is generally higher in adult males than females [27]. AngII infusion induces hypertension in male but not in female rodents, indicating that estrogen protection shifts the balance from AngII to Ang(1-7) pathways [27].

### 3.4. Angiotensin-Converting Enzyme 2 (ACE2) and Ang(1-7)

The disclosure of ACE2 has a potential therapeutic role in RAS modulation [33]. This ACE homologue ACE2 monocarboxypeptidase is the main source of the vasodilator and natriuretic peptide Ang(1-7) [13,26]. ACE2 can form Ang (1–7) directly or indirectly from either the AngI or from AngII [13]. ACE2 preferentially removes carboxy-terminal amino acids from substrates including AngII, AngI, and apelin [25]. Ang(1-7) mediate its effect through the Mas receptor (MasR), a G protein-coupled receptor [34].

In contrast with ACE, ACE2 does not convert AngI to AngII and is not inhibited by ACE inhibitors. ACE2 increases degradation of AngII and reduces AngII formation by competitively stimulating alternative pathways for AngI degradation [29].

ACE2 expression and activity is found in multiple tissues including heart, kidney, liver, skeletal muscle, adipose, and pancreas [27]. ACE2 expression is upregulated in the serum, kidney, pancreas, and liver of male and female diabetic rodents suggesting a compensatory protective mechanism [27]. Levels of ACE2, Ang(1-7) production, and MasR are found increased in splanchnic vessels from cirrhotic patients and rats compared to healthy controls [26]. ACE2 is now thought to be a negative regulator of the RAS and, in the liver, this enzyme generates Ang(1-7) that may inhibit experimental liver fibrosis [2,35]. In many cases, the second axis ACE2/Ang (1-7)/MasR appears to counter-regulate the effects of the classical RAS axis [13].

ACE2 is upregulated in chronic liver injury in order to limit fibrogenesis by the cleavage of AngII [35]. Hepatic ACE2 overexpression would be expected to produce dual benefits, breakdown of the potent profibrotic peptide AngII and generation of the anti-fibrotic peptide Ang(1-7) within the liver [36]. Ang(1-7) peptide reduces matrix formation in cultured rat hepatic stellate cells (HSCs) which leads to pronounced improvement in hepatic fibrosis [34]. Ang(1-7) tends to reduce HSC activation and pro-inflammatory and pro-fibrotic mediators [36]. Ang(1-7) infusion inhibited the expression of the potent profibrotic cytokine TGFβ1 in the early development of fibrosis [36]. ACE2 knock out mice show increased serum and tissue AngII and develop spontaneous glomerulosclerosis with increased deposition of fibrillary collagen [35]. Blockade of Mas receptor enhances liver fibrosis by increasing the liver content of collagen and TGFβ1 [5].

The non-classical RAS, through the ACE2, mediates the entry of the etiological agent of COVID-19 (SARS-CoV-2) into cells. This may induce a reduction in ACE2 and an imbalance between angiotensins in favor of AngII that may be responsible for the lung and heart damage [20]. In another study, ACE2 has been identified as a functional receptor for the severe acute respiratory syndrome SARS-CoV in vitro and in vivo. The SARS-CoV receptor ACE2 is expressed in the lungs of healthy and diseased humans. SARS Spike-protein-mediated ACE2 downregulation appears to contribute to the severity of lung failure [37].

## 4. Crosstalk between RAS, Liver Lipid and Carbohydrate Metabolism

RAS plays a critical role in lipid and carbohydrate metabolism and also is involved in prevention, diagnosis and treatment of many diseases. Alterations in RAS functioning is related to hepatic and diabetic disorders such as type 2 diabetes mellitus (T2DM), obesity, and NAFLD [6,33].

RAS overactivity represented by the increase in local AngII and activation of AT1R may lead to liver dysfunction promoting inflammation, liver steatosis and lipid metabolism dysregulation [38]. In metabolic disease of the liver it has been shown there is an imbalance between ACE/AngII/AT1 and ACE2/Ang(1-7)/Mas [31]. Several studies have suggested that local hepatic ACE2 overexpression and blockers of AngII and ATR1 can be used in the prevention and treatment of diabetes, insulin resistance, glucose homeostasis, hepatic steatosis, NAFLD, liver fibrosis and other metabolic disorders [2,16,33,34,39,40]. In fact, Ang(1-7) has a marked effect in decreasing liver gluconeogenesis, and to improve carbohydrate metabolism.

**Figure 5:**
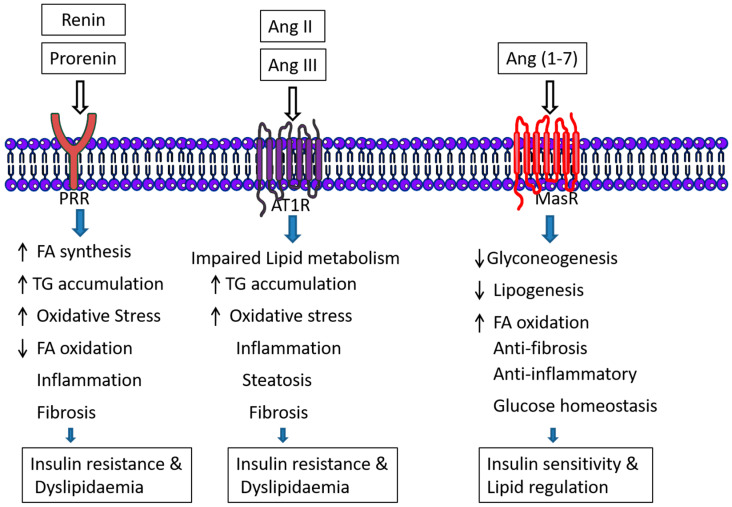
Effect of RAS in lipid and carbohydrate metabolism. Two RAS arms, ACE/AngII/AT1 and ACE2/Ang(1-7)/MasR, act antagonistically on lipid and carbohydrate metabolism. PRR also accelerates the FA synthesis and tricarboxylic acid cycle (TCA).

On the contrary, several studies supported that increased levels of AngII accelerate inflammation, oxidative stress and vascular injury in hepatic patients and lead to liver fibrosis [13]. Inappropriately elevated AngII and over-activation of its target receptor, AT1, contribute to impaired hepatic lipid metabolism and development of fatty liver and insulin resistance [40]. A large number of studies have shown that insulin resistance linked to dysregulation of lipid metabolism and insulin resistance is a high-risk factor for hepatocellular carcinoma (HCC); it can increase the risk of HCC by 2–3 times [41]. Interestingly, RAS is also involved in mitigating NAFLD by reducing liver lipogenesis, increasing fatty acid oxidation, and inhibiting gluconeogenesis in rodents. The mechanism behind it is the yet to be mentioned opposing effects of the two arms of RAS, the inhibition of the ACE/AngII/AT1R axis by AT1R blockers or by activation of the ACE2/Ang1-7/MasR axis [6] (Figure 5). The increased accumulation of intrahepatic lipids may drive local liver inflammation and steatohepatitis, which when maintained over time may evolve to liver fibrosis and lastly cirrhosis. Some studies have shown that high circulating ACE level may be used as a marker of evolving fibrosis in hepatitis B patients [39]. Liver fibrosis progression is accompanied by angiogenesis [42] which is in part regulated by RAS components [43,44]. The renin–angiotensin system (RAS) plays an essential role in developmental and pathologic blood vessel growth; angiotensin II possesses proangiogenic activity. Studies in mouse models show that ACE inhibitors reduce tumor growth by blocking angiogenesis [44].

Hepatic fibrosis is related to AngII/AT1 activation, meanwhile Ang(1-7) exhibited beneficial effects against hepatic fibrosis [33]. The ACE 2/Ang(1-7)/Mas axis activates Akt signaling to improve hepatic steatosis [45]. AngII accelerates NADPH oxidase and increases reactive oxygen species (ROS) generation through the angiotensin type 1 (AT1) receptor. ROS activates the NFᴋB to increase transcription of cytokines (TNFᾳandIL-6) that leads to inhibiting insulin signaling [46]. AngII acts by amplifying the general inflammatory response that follows the chronic liver injury, inducing ROS generation and inflammatory cytokines [16].

Ang(1-7) has been involved in decreasing liver gluconeogenesis, and the Mas receptor is an important component of the insulin receptor signalling [33].

Given all that is mentioned above, the RAS plays a central role in cellular responses to insulin (insulin sensitivity) [16]. Chronic impaired tissue responses to insulin are associated with hyperglycemia and hyperlipidemia. Moreover, boost islet damage leads to worse IR that in turn dysregulates glycolipid metabolism by impaired endocrine pancreatic function [16].

## 5. Gender in RAS, Liver Lipid and Carbohydrate Metabolism

In previous studies, it is concluded that women’s metabolism substantially differs from men’s [10]. Several studies have shown sex differences in body composition and visceral and subcutaneous fat distribution. Females have more subcutaneous adipose tissue compared with a greater visceral adipose distribution in males [47]. Excess fat stores in the visceral tissues, is associated with higher TG levels in the liver [47], and has a higher contribution to fatty acid (FA) delivery to the liver compared to adipose from subcutaneous fat [48]. These sex differences in adipose distribution have been linked to metabolic health having, the females currently show a more favorable lipid and glucose metabolism profile compared with males [27]. Sex differences in metabolic traits such as obesity, diabetes, and cardiovascular disease have been widely described in mice, humans, and other species, with females generally exhibiting more beneficial metabolic profiles than males [8].

NAFLD is also more frequent in men than women, and men exhibit more severe symptoms when NAFLD is present [8]. Interestingly, as shown in other reports, hormones, gene expression differences from the X and Y chromosomes and genetic background may be responsible for the differences in the levels of enzymes and lipids between both sexes [8]. According to one report, the ACE2 gene is located on the X chromosome, with females generally having higher ACE2 activity [27]. Previous studies focused on the vasoconstrictor arm of the RAS (ACE/AngII/AT1R) in rodents, illustrating that females are protected from cardiovascular and metabolic disorders produced by AGT, renin, and angiotensin II, as compared to males. By involving estrogen downregulating AngII and upregulating Ang(1-7) pathways, female animal models appear to maintain higher circulating Ang(1-7) levels, which protect them from hypertension and metabolic complications induced by AngII and AT1R activation [27]. It has been reported that AT2R deletion exerts a negative impact on insulin sensitivity and glucose homeostasis in female mice compared to males [49].

Several RAS components are differentially expressed in females, with estrogen downregulating AngII and upregulating Ang(1-7) pathways [27]. Accordingly, sex-dependent differences in RAS responses observed between men and women might be linked to the levels of sex hormones [14]. Activation of the vasodilator branch of RAS, including Ang(1-7), ACE2, and mas receptors, has been attributed to the exertion of beneficial effects on glucose homeostasis and lipid metabolism in male animal models, despite there being no sufficient data available in females [27].

## 6. Effect of Perinatal Undernutrition on RAS, Liver Lipid and Carbohydrate Metabolism

**Figure 6:**
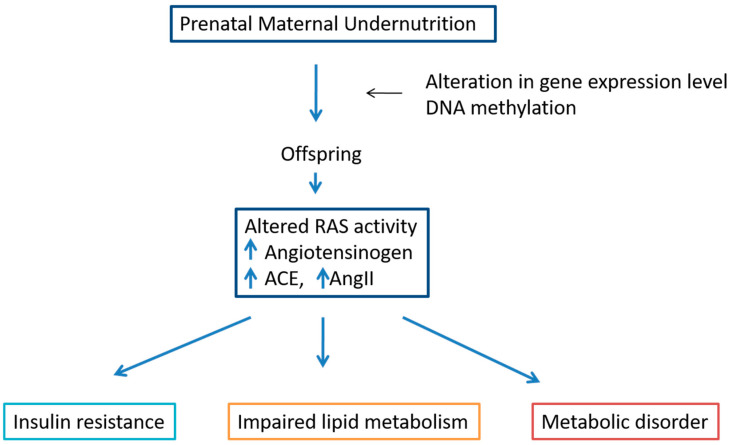
Effect of prenatal undernutrition on RAS. Prenatal undernutrition has linked with substantial changes in RAS components of offspring resulting in impaired lipid and carbohydrate metabolism.

Undernutrition is an increasingly common problem; about 800 million people globally suffer from hunger, while studies regarding the effects of undernutrition on various aspects of human life are insufficient. The effects of maternal undernutrition, calorie restriction, and deficiencies in macro- and micronutrients may have long term effects on hepatic lipid accumulation and metabolism regulation. Undernutrition in mothers creates immediate health problems, not only in women and newborns, but its impact may also be transmitted to later generations. Maternal nutrient deficiency affects gene expression levels by epigenetic mechanisms such as DNA methylation [50] (Figure 6). Consequently, prenatal undernutrition impairs lipid metabolism by altering the mRNA levels of key genes responsible for lipid homeostasis [51]. Prenatal undernutrition has been associated with substantial changes in endocrine functions in adulthood [7]. Maternal calorie deficiency clearly alters lipid metabolism in offspring, and a tight link was observed between low birth weight and NAFLD in those individuals [51]. Caloric restriction in the perinatal period is also related to type 2 diabetes, glucose intolerance and obesity [52]. It has been shown in many previous studies that undernourishment may alter the RAS components’ level, which in turn generates many metabolic disorders and diseases. AGT and ACE expression are increased in adipose tissue of malnourished individuals, promoting adiposity and lipid accumulation [53].

In intrauterine undernourished rats, over-activity of chymase may result in increased intrarenal AngII production, which contributes to the development of hypertension [54]. Low protein intake in early life enhances RAS activity, associated with metabolic changes, cardiovascular impairment and high blood pressure during adulthood [55]. Several nutritional factors including low-protein diet during early life lead to a predisposition toward dysregulation of RAS components [56]. Fetal and lactation undernutrition result in increased AngII and alterations in RAS cardiac AT1, AT2, MasR and MrgD receptors in male pups [57]. Maternal food restriction may affect many brain areas; the total number of neurons and granule cells are remarkably reduced [58]. Moderate food restriction modulates RAS components and cytokine expression in mice and improves the metabolism [53]. In utero environmental changes such as the antennal hypoxia reduce the ACE expression in fetal lungs, leading to the upregulation of ACE-2 at both transcriptional and translational levels. In addition, in utero environment conditions promote epigenetic changes which modify the expression of several genes of the pulmonary RAS [59].

## 7. GLP-1R Agonists Regulate the Liver Lipids and Carbohydrate Metabolism

The RAS plays a key role in regulating glucose and lipid metabolism and liver function [6]. ACE2 deficient mice have shown significant liver metabolic disorders [6]. Glucagon-like peptide 1 (GLP-1) is a potent incretin hormone produced in the L-cells of the distal ileum and colon. Previous studies demonstrate that concentration of biologically active incretin hormone is lower in NAFLD patients as compared to healthy individuals and GLP-receptor agonists can improve liver injury, lipid metabolism and metabolic disorder in patients with liver disease [60]. One previous study shows that G protein-coupled receptor GLP-1R is present on human hepatocytes [61]. In addition, immunohistochemistry has shown that the GLP-1 receptor is present in the lipid microdomains of hepatocytes with steatosis besides the pancreatic islet cells, lung, brain, kidney and adipose tissue of animals and humans [62].

One of the meta-analyses shows that GLP-1 receptor agonists significantly improve the liver fat contents [60]. Liraglutide is a GLP-1 receptor agonist which has been shown to have a potent regulatory effect in the RAS biological actions [63]. In the β cells, the GLP-1 agonist receptor enhances insulin secretion in a glucose-dependent manner by accelerating the metabolism of glucose that increase the ATP generation, which result in closure of the ATP-sensitive KC channels and the subsequent influx of calcium [63].

Liraglutide ameliorated NAFLD and promoted glucose metabolism in mice livers [6,64] and it is able to induce a marked increase in the expression levels of ACE-2 in the lungs of animal models in different experimental conditions [59]. It has an antagonistic effect on the ACE/AngII/AT1R axis and a positive impact on the ACE2/Ang(1-7)/MasR axis [6,59] and is mediated through the PI3K/AKT pathway [6].

ACE2 knockout increases lipid accumulation and the severity of liver steatosis [6]. Liraglutide treatment activates the ACE2/Ang1-7/Mas axis, increases fatty acid oxidation gene expression, suppression of gluconeogenesis, and inflammation in the liver associated with NF-kB activation, which is impaired in ACE2-KO mice [6]. Liraglutide downregulates ACE and AT1R while further upregulating ACE2 and MasR expression. These findings indicate that liraglutide plays a dual regulatory role on the two arms of the RAS [6]. In patients with type 2 diabetes mellitus, GLP-1 promotes a significant increase in insulin sensitivity in both skeletal muscle and adipose tissue, with improvements in insulin-mediated glucose uptake [65].

**Figure 7:**
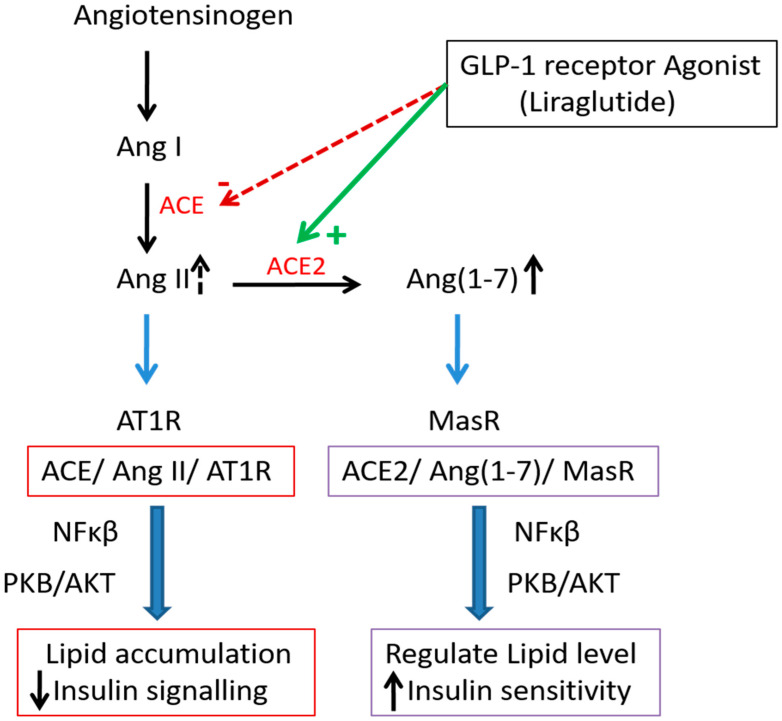
Role of GLP-1 receptor agonist liraglutide to regulate lipid and carbohydrate metabolism. Liraglutide lightly activates ACE/AngII/AT1 arm and markedly stimulates ACE2/Ang(1-7)/MasR.

Previous studies have demonstrated that GLP-1R activation might play a role in regulation of RAS components in different organs [66]. Liraglutide modulates the pulmonary angiotensins and their receptors and increases the expression of key surfactant proteins in the lungs [60]. Liraglutide is able to modulate the RAS expression levels in the lungs, especially stimulating the expression of ACE-2 [59] (Figure 7), that leads to increased production of Ang(1-7), activating the MasR [66]. Ang(1-7) reduces lung fibrosis and pulmonary arterial hypertension and activates pathways that promote lung homeostasis [59]. The GLP-1 receptor agonist improves myocardial glucose utilization in heart failure by activating adenylate cyclase and phosphorylates the cAMP-dependent protein kinase that enhances insulin-dependent signaling, increases glucose uptake and provides other cardioprotective actions [65]. In addition, dipeptidyl peptidase-4 inhibition, an enzyme that degrades endogenous GLP-1 and GLP-1R activation, can reverse cardiac fibrosis induced by angiotensin II by restoring angiotensin 2 type 2 receptor (ATR2)/ACE2 imbalance [66]. This may represent a potential new mechanism by which liraglutide can improve lipid and carbohydrate metabolism.

## 8. Discussion and Concluding Remarks

Liver is a metabolically active organ, where carbohydrates may be transformed into fatty acids (FA), which can be esterified into TG for storage, or for secretion into lipoproteins that will be used by other tissues [11]. Liver diseases are increasing in the modern world and are becoming a serious public health problem. Non-alcoholic fatty liver disease (NAFLD) is one of the most prevalent chronic liver diseases worldwide [67]. NAFLD can be accompanied by various metabolic disorders, including obesity, diabetes and hyperlipidemia. The abnormal metabolism of carbohydrates and fats due to an imbalance in hepatic metabolism can result in insulin resistance, as the liver is a preferential target organ for insulin-regulatory effects. An imbalance in hepatic metabolism may result in disease which causes hepatic dysfunction [68].

Prenatal undernutrition may exert a long-term effect on lipid metabolism not only in offspring but also transmitted in later generations by changing gene expression levels [69]. There are several clues that offspring’s lipid metabolism and steatosis are promoted by maternal undernutrition [51] altering the mRNA level of key genes responsible for lipid homeostasis [69]. Intrauterine growth restriction (IUGR) may result in increased intrarenal AngII production by overactivity of chymase [50]. The GLP-1 receptor agonist acts neuroprotectively that minimizes the harmful effects of maternal food restriction [58]. AngII has been shown to modulate lipolysis, lipogenesis and adipocyte differentiation [38]. AngII also, via AngII receptor type 1 (AT1), stimulates cholesterol synthesis, low-density lipoprotein cholesterol (LDL-C) oxidation and its incorporation into the vascular wall [38].

Several studies show a link between liver diseases and over-activation of RAS components and ACE/AT1 receptor, and it is also demonstrated that ACE/AngII/AT1 axis inhibitors block AngII production and its effects, preventing the development of hepatic fibrosis and other liver diseases [1,3]. RAS is known to be altered during the pathogenesis of liver fibrosis [3] and the new therapeutic group called RAS blockers, and the ACE inhibitors telmisartan, losartan and perindopril ameliorate the hepatic fibrosis in ACE/Ang II/AT1 while increasing ACE2/Ang(1-7)/Mas activation [3]. Chronic blockade of AT1 protects the liver from triacylglycerol (TAG) accumulation, particularly during a glucose load [40]. Some clinical studies indicate that a reduction in hyperlipidemia and RAS blockade may have a synergistic effect [38]. RAS inhibitors that are used as classical antihypertensive drugs can lessen the occurrence of T2DM [6] and improve NAFLD and liver injury [3].

The discovery of ACE2 has reopened investigation into the potential therapeutic role of RAS modulation [6]. Globally, modulation of RAS is recognized as a first-line strategy in the treatment of hypertension and cardiovascular disease [70]. ACE inhibitors and ARBs are widely used to treat cardiovascular diseases [18]. Previous studies demonstrate RAS has a key link with NAFLD and also indicate that inhibiting the ACE/AngII/AT1R axis or activating the ACE2/Ang1-7/Mas axis may represent effective targets for NAFLD treatment [6]. The activation of Ang(1-7) pathways is also an attractive target to improve glucose homeostasis, lipid metabolism and energy balance in male animal models, and female comparison studies and clinical data related to metabolic outcomes are eagerly awaited [27].

Thus, based upon findings, it may be concluded that the use of classical RAS axis (ACE/AngII/AT1) inhibitors associated with non-classical RAS axis (ACE2/Ang(1-7)/Mas) activation is a promising new strategy serving as a novel therapeutic regimen to prevent and treat chronic liver diseases as well as acute liver injury [3,5,6]. The GLP-1 analogue liraglutide modulates both axes of RAS, especially augmenting the expression levels of ACE-2, which in turn drive the reduction in circulating AngII and the marked increase in Ang(1-7) [6]. However, the factors regulating the expression and activity of the RAS are still largely unknown, and extensive research gaps remain [6]. There is a lack of studies carried out in females, especially studies related to metabolism in respect of RAS, which is clearly different from males. The identification of sex-specific mechanisms underlying the metabolic effects of the RAS, as well as the beneficial effects of therapies targeting the RAS, remains an active area of research [27].

## References

[1] de Silva A.C.S., Miranda A.S., Rocha N.P., Teixeira A.L. (2017). Renin angiotensin system in liver diseases: Friend or foe?. World J. Gastroenterol..

[2] Lastra-Lastra G., Sowers J.R., Restrepo-Erazo K., Manrique-Acevedo C., Lastra-González G. (2009). Role of aldosterone and angiotensin II in insulin resistance: An update. Clin. Endocrinol..

[3] de Macêdo S.M., Guimarães T.A., Feltenberger J.D., Santos S.H.S. (2014). The role of renin-angiotensin system modulation on treatment and prevention of liver diseases. Peptides.

[4] Ramadori G., Saile B. (2004). Inflammation, damage repair, immune cells, and liver fibrosis: Specific or nonspecific, this is the question. Gastroenterology.

[5] Pereira R.M., dos Santos R.A.S., Dias F.L.D.C., Teixeira M.M., e Silva A.C.S. (2009). Renin-angiotensin system in the pathogenesis of liver fibrosis. World J. Gastroenterol..

[6] Yang M., Ma X., Xuan X., Deng H., Chen Q., Yuan L. (2020). Liraglutide Attenuates Non-Alcoholic Fatty Liver Disease in Mice by Regulating the Local Renin-Angiotensin System. Front. Pharmacol..

[7] Khanal P., Nielsen M.O. (2017). Impacts of prenatal nutrition on animal production and performance: A focus on growth and metabolic and endocrine function in sheep. J. Anim. Sci. Biotechnol..

[8] Krishnan K.C., Mehrabian M., Lusis A.J. (2018). Sex differences in metabolism and cardiometabolic disorders. Curr. Opin. Lipidol..

[9] Taskin E., Guven C., Tolekova A.N. (2017). Local Renin angiotensin System at liver and crosstalk with hepatic diseases. Renin-Angiotensin System.

[10] Mittendorfer B. (2005). Sexual Dimorphism in Human Lipid Metabolism. J. Nutr..

[11] Choi G.Y., Tosh D.N., Garg A., Mansano R., Ross M.G., Desai M. (2007). Gender-specific programmed hepatic lipid dysregulation in intrauterine growth-restricted offspring. Am. J. Obstet. Gynecol..

[12] Thiriet M. (2018). Hyperlipidemias and Obesity. Vasculopathies.

[13] Santos R.A.S., Sampaio W.O., Alzamora A.C., Motta-Santos D., Alenina N., Bader M., Campagnole-Santos M.J. (2018). The ACE2/Angiotensin-(1–7)/MAS Axis of the Renin-Angiotensin System: Focus on Angiotensin-(1–7). Physiol. Rev..

[14] Pidikova P., Svitok P., Herichova I. (2019). Sex and salt intake dependent renin-angiotensin plasticity in the liver of the rat. Endocr. Regul..

[15] Neo J.H., I Ager E.I., Angus P.W., Zhu J., Herath C.B., Christophi C. (2010). Changes in the renin angiotensin system during the development of colorectal cancer liver metastases. BMC Cancer.

[16] Georgescu E.F. (2008). Angiotensin receptor blockers in the treatment of NASH/NAFLD: Could they be a first-class option?. Adv. Ther..

[17] Kon Y., Endoh D., Yamashita T., Watanabe T. (1998). Expression of Renin in the Rat Liver. Anat. Histol. Embryol..

[18] Tomita S., Tomita N., Yamada T., Zhang L., Kaneda Y., Morishita R., Ogihara T., Dzau V.J., Horiuchi M. (1999). Transcription factor decoy to study the molecular mechanism of negative regulation of renin gene expression in the liver in vivo. Circ. Res..

[19] Koh S.L., Ager E., Christophi C. (2010). Liver regeneration and tumour stimulation: Implications of the renin-angiotensin system. Liver Int..

[20] Mascolo A., Scavone C., Rafaniello C., De Angelis A., Urbanek K., di Mauro G., Cappetta D., Berrino L., Rossi F., Capuano A. (2021). The Role of Renin-Angiotensin-Aldosterone System in the Heart and Lung: Focus on COVID-19. Front. Pharmacol..

[21] Herrmann H.C., Dzau V.J. (1983). The Feedback Regulation of Angiotensinogen Production by Components of the Renin-Angiotensin System. Circ. Res..

[22] Klett C., Nobiling R., Gierschik P., Hackenthal E. (1993). Angiotensin II stimulates the synthesis of angiotensinogen in hepatocytes by inhibiting adenylyl cyclase activity and stabilizing angiotensinogen mRNA. Int. J. Biochem. Mol. Biol..

[23] Sernia C., Reid I.A. (1980). Stimulation of angiotensinogen production: A dose-related effect of angiotensin II in the conscious dog. Am. J. Physiol. Metab..

[24] Lai H.-S., Lin W.-H., Lai S.-L., Lin H.-Y., Hsu W.-M., Chou C.-H., Lee P.-H. (2013). Interleukin-6 Mediates Angiotensinogen Gene Expression during Liver Regeneration. PLoS ONE.

[25] Hsieh Y.-C., Lee K.-C., Lei H.-J., Lan K.-H., Huo T.-I., Lin Y.-T., Chan C.-C., Schnabl B., Huang Y.-H., Hou M.-C. (2021). (Pro)renin Receptor Knockdown Attenuates Liver Fibrosis Through Inactivation of ERK/TGF-β1/SMAD3 Pathway. Cell. Mol. Gastroenterol. Hepatol..

[26] Sansoè G., Aragno M., Wong F. (2019). Pathways of hepatic and renal damage through non-classical activation of the renin-angiotensin system in chronic liver disease. Liver Int..

[27] White M.C., Fleeman R., Arnold A.C. (2019). Sex differences in the metabolic effects of the renin-angiotensin system. Biol. Sex Differ..

[28] Ren L., Sun Y., Lu H., Ye D., Han L., Wang N., Daugherty A., Li F., Wang M., Su F. (2018). (Pro)renin Receptor Inhibition Reprograms Hepatic Lipid Metabolism and Protects Mice From Diet-Induced Obesity and Hepatosteatosis. Circ. Res..

[29] Wang C.-H., Li F., Takahashi N. (2010). The renin angiotensin system and the metabolic syndrome. Open Hypertens. J..

[30] Shim K.Y., Eom Y.W., Kim M.Y., Kang S.H., Baik S.K. (2018). Role of the renin-angiotensin system in hepatic fibrosis and portal hypertension. Korean J. Intern. Med..

[31] Saber S. (2018). Angiotensin II: A key mediator in the development of liver fibrosis and cancer. Bull. Natl. Res. Cent..

[32] Jonsson J.R., Clouston A.D., Ando Y., Kelemen L.I., Horn M.J., Adamson M.D., Purdie D.M., Powell E.E. (2001). Angiotensin-Converting Enzyme Inhibition Attenuates the Progression of Rat Hepatic Fibrosis. Gastroenterology.

[33] Tiao M.-M., Lin Y.-J., Yu H.-R., Sheen J.-M., Lin I.-C., Lai Y.-J., Tain Y.-L., Huang L.-T., Tsai C.-C. (2018). Resveratrol ameliorates maternal and post-weaning high-fat diet-induced nonalcoholic fatty liver disease via renin-angiotensin system. Lipids Health Dis..

[34] Rajapaksha I., Gunarathne L., Angus P., Herath C. (2021). Update on New Aspects of the Renin-Angiotensin System in Hepatic Fibrosis and Portal Hypertension: Implications for Novel Therapeutic Options. J. Clin. Med..

[35] Österreicher C.H., Taura K., De Minicis S., Seki E., Penz-Österreicher M., Kodama Y., Kluwe J., Schuster M., Oudit G.Y., Penninger J.M. (2009). Angiotensin-converting-enzyme 2 inhibits liver fibrosis in mice. Hepatology.

[36] Rajapaksha I.G., Gunarathne L.S., Asadi K., Cunningham S.C., Sharland A., Alexander I.E., Angus P.W., Herath C.B. (2019). Liver-Targeted Angiotensin Converting Enzyme 2 Therapy Inhibits Chronic Biliary Fibrosis in Multiple Drug-Resistant Gene 2-Knockout Mice. Hepatol. Commun..

[37] Kuba K., Imai Y., Rao S., Jiang C., Penninger J.M. (2006). Lessons from SARS: Control of acute lung failure by the SARS receptor ACE2. J. Mol. Med..

[38] Pizoń T., Rajzer M., Wojciechowska W., Wach-Pizoń M., Drożdż T., Wróbel K., Gruszka K., Rojek M., Kameczura T., Jurczyszyn A. (2018). The relationship between plasma renin activity and serum lipid profiles in patients with primary arterial hypertension. J. Renin-Angiotensin-Aldosterone Syst..

[39] Zhu Q., Li N., Li F., Zhou Z., Han Q., Lv Y., Sang J., Liu Z. (2016). Therapeutic effect of renin angiotensin system inhibitors on liver fibrosis. J. Renin-Angiotensin-Aldosterone Syst..

[40] Utba R.M., Hussain S.A., Fadhil A.A., Ahmed A. (2016). Effect of Azilsartan, Aliskirenor Their Combination on Body Weightand Adipogenesis of High-fat Diet Induced Non-alcoholic Fatty Liver Disease in Rats. Am. J. Pharmacol. Sci..

[41] Zhang H.-F., Gao X., Wang X., Chen X., Huang Y., Wang L., Xu Z.-W. (2021). The mechanisms of renin–angiotensin system in hepatocellular carcinoma: From the perspective of liver fibrosis, HCC cell proliferation, metastasis and angiogenesis, and corresponding protection measures. Biomed. Pharmacother..

[42] Lei L., Mourabit H.E., Housset C., Cadoret A., Lemoinne S. (2021). Role of Angiogenesis in the Pathogenesis of NAFLD. J. Clin. Med..

[43] Heffelfinger S.C. (2007). The Renin Angiotensin System in the Regulation of Angiogenesis. Curr. Pharm. Des..

[44] Khakoo A.Y., Sidman R.L., Pasqualini R., Arap W. (2008). Does the Renin-Angiotensin System Participate in Regulation of Human Vasculogenesis and Angiogenesis?. Cancer Res..

[45] Cao X., Yang F., Shi T., Yuan M., Xin Z., Xie R., Li S., Li H., Yang J.-K. (2016). Angiotensin-converting enzyme 2/angiotensin-(1–7)/Mas axis activates Akt signaling to ameliorate hepatic steatosis. Sci. Rep..

[46] du Toit E.F., Donner D.G. (2012). Myocardial Insulin Resistance: An Overview of Its Causes, Effects, and Potential Therapy. Insulin Resistance.

[47] Magkos F., Mittendorfer B. (2009). Gender Differences in Lipid Metabolism and the Effect of Obesity. Obstet. Gynecol. Clin. N. Am..

[48] Palmisano B.T., Zhu L., Eckel R.H., Stafford J.M. (2018). Sex differences in lipid and lipoprotein metabolism. Mol. Metab..

[49] Quiroga D.T., Miquet J.G., González L., Sotelo A.I., Muñoz M.C., Geraldes P.M., Giani J.F., Dominici F.P. (2019). Mice lacking angiotensin type 2 receptor exhibit a sex-specific attenuation of insulin sensitivity. Mol. Cell. Endocrinol..

[50] Chmurzynska A. (2010). Fetal programming: Link between early nutrition, DNA methylation, and complex diseases. Nutr. Rev..

[51] Lecoutre S., Montel V., Vallez E., Pourpe C., Delmont A., Eury E., Verbanck M., Dickes-Coopman A., Daubersies P., Lesage J. (2019). Transcription profiling in the liver of undernourished male rat offspring reveals altered lipid metabolism pathways and predisposition to hepatic steatosis. Am. J. Physiol. Metab..

[52] Ma H., Sales V.M., Wolf A.R., Subramanian S., Matthews T.J., Chen M., Sharma A., Gall W., Kulik W., Cohen D.E. (2017). Attenuated Effects of Bile Acids on Glucose Metabolism and Insulin Sensitivity in a Male Mouse Model of Prenatal Undernutrition. Endocrinology.

[53] Pinheiro T.D.A., Barcala-Jorge A.S., Andrade J.M.O., Ferreira E.C.N., Crespo T.S., Batista-Jorge G.C., Vieira C.A., Lelis D.D.F., Paraiso A.F., Pinheiro U.B. (2017). Obesity and malnutrition similarly alter the renin–angiotensin system and inflammation in mice and human adipose. J. Nutr. Biochem..

[54] Chou H.-C., Wang L.-F., Lu K.-S., Chen C.-M. (2008). Effects of maternal undernutrition on renal angiotensin II and chymase in hypertensive offspring. Acta Histochem..

[55] Silva F.C.D.S., De Menezes R.C., Chianca D.A. (2015). The implication of protein malnutrition on cardiovascular control systems in rats. Front. Physiol..

[56] Hsu C.-N., Tain Y.-L. (2018). The Double-Edged Sword Effects of Maternal Nutrition in the Developmental Programming of Hypertension. Nutrients.

[57] Rodríguez-Rodríguez P., Vieira-Rocha M., Quintana-Villamandos B., Monedero-Cobeta I., Prachaney P., de Pablo A.L., González M., Morato M., Diniz C., Arribas S. (2021). Implication of RAS in Postnatal Cardiac Remodeling, Fibrosis and Dysfunction Induced by Fetal Undernutrition. Pathophysiology.

[58] Diz-Chaves Y., Toba L., Fandiño J., González-Matías L.C., Garcia-Segura L.M., Mallo F. (2018). The GLP-1 analog, liraglutide prevents the increase of proinflammatory mediators in the hippocampus of male rat pups submitted to maternal perinatal food restriction. J. Neuroinflammation.

[59] Fandiño J., Vaz A.A., Toba L., Romaní-Pérez M., Matías L.C.G., Mallo F., Diz-Chaves Y. (2018). Liraglutide Enhances the Activity of the ACE-2/Ang(1–7)/Mas Receptor Pathway in Lungs of Male Pups from Food-Restricted Mothers and Prevents the Reduction of SP-A. Int. J. Endocrinol..

[60] Dai Y., He H., Li S., Yang L. (2021). Comparison of the Efficacy of Glucagon-Like Peptide-1 Receptor Agonists in Patients with Metabolic Associated Fatty Liver Disease: Updated Systematic Review and Meta-Analysis. Endocrinology.

[61] Gupta N.A., Mells J., Dunham R.M., Grakoui A., Handy J., Saxena N.K., Anania F.A. (2010). Glucagon-like peptide-1 receptor is present on human hepatocytes and has a direct role in decreasing hepatic steatosis in vitro by modulating elements of the insulin signaling pathway. Hepatology.

[62] Yokomori H., Ando W. (2020). Spatial expression of glucagon-like peptide 1 receptor and caveolin-1 in hepatocytes with macrovesicular steatosis in non-alcoholic steatohepatitis. BMJ Open Gastroenterol..

[63] Seghieri M., Christensen A.S., Andersen A., Solini A., Knop F.K., Vilsbøll T. (2018). Future Perspectives on GLP-1 Receptor Agonists and GLP-1/glucagon Receptor Co-agonists in the Treatment of NAFLD. Front. Endocrinol..

[64] Capuani B., Pacifici F., Della-Morte D., Lauro D. (2018). Glucagon Like Peptide 1 and MicroRNA in Metabolic Diseases: Focusing on GLP1 Action on miRNAs. Front. Endocrinol..

[65] Margulies K.B., Anstrom K.J., Hernandez A.F., Redfield M.M., Shah M.R., Braunwald E., Cappola T.P. (2014). GLP-1 Agonist Therapy for Advanced Heart Failure With Reduced Ejection Fraction. Circ. Heart Fail..

[66] Fandiño J., Toba L., González-Matías L.C., Diz-Chaves Y., Mallo F. (2020). GLP-1 receptor agonist ameliorates experimental lung fibrosis. Sci. Rep..

[67] Estes C., Razavi H., Loomba R., Younossi Z., Sanyal A.J. (2018). Modeling the epidemic of nonalcoholic fatty liver disease demonstrates an exponential increase in burden of disease. Hepatology.

[68] Ding H.-R., Wang J.-L., Ren H.-Z., Shi X.-L. (2018). Lipometabolism and Glycometabolism in Liver Diseases. BioMed Res. Int..

[69] Nowacka-Woszuk J., Madeja Z.E., Chmurzynska A. (2017). Prenatal caloric restriction alters lipid metabolism but not hepatic Fasn gene expression and methylation profiles in rats. BMC Genet..

[70] Hamet P. (2005). The Renin-Angiotensin System: Where Do We Stand, and What Is the Future?. Am. J. Hypertens..

